# Versatile control of the CDC48 segregase by the plant UBX-containing (PUX) proteins

**DOI:** 10.1016/j.csbj.2021.05.025

**Published:** 2021-05-14

**Authors:** Junrui Zhang, Alexandra I. Vancea, Umar F. Shahul Hameed, Stefan T. Arold

**Affiliations:** a King Abdullah University of Science and Technology (KAUST), Computational Bioscience Research Center (CBRC), Biological and Environmental Science and Engineering (BESE), Thuwal 23955-6900, Saudi Arabia; b Centre de Biochimie Structurale, CNRS, INSERM, Université de Montpellier, 34090 Montpellier, France

**Keywords:** CDC48, Cofactors, PUX proteins, Segregase, LDAD, ERAD, Autophagy, Structure, Adaptor

## Abstract

In plants, AAA-adenosine triphosphatase (ATPase) Cell Division Control Protein 48 (CDC48) uses the force generated through ATP hydrolysis to pull, extract, and unfold ubiquitylated or sumoylated proteins from the membrane, chromatin, or protein complexes. The resulting changes in protein or RNA content are an important means for plants to control protein homeostasis and thereby adapt to shifting environmental conditions. The activity and targeting of CDC48 are controlled by adaptor proteins, of which the plant ubiquitin regulatory X (UBX) domain-containing (PUX) proteins constitute the largest family. Emerging knowledge on the structure and function of PUX proteins highlights that these proteins are versatile factors for plant homeostasis and adaptation that might inspire biotechnological applications.

## 1 Introduction

Organisms need to adapt rapidly in response to molecular cues or changing conditions. Although this capacity to react is essential to all life forms, it is particularly crucial to plants, given that they cannot adapt by changing their location. At a cellular level, such adaptation generally requires the modification of the protein or RNA composition in specific subcellular loci. This adaptation is most effectively and rapidly achieved via changes in the protein/RNA expression profile, concomitant with an active and selective relocation or degradation of proteins from these loci [1], [2].

The cell division control protein 48 (CDC48) constitutes the core of a versatile multiprotein complex that can do both, alter protein/RNA expression and extract proteins from their environment for reuse or degradation [3], [4]. CDC48 is a type II adenosine triphosphatase (ATPase) associated with diverse cellular activities (AAA ATPase). Five isoforms of CDC48 have been identified in *Arabidopsis thaliana*: CDC48A, B and C are close paralogues, whereas CDC48D and E are more distantly related [5]. Of these CDC48A is the best characterized and possibly most important isoform in *Arabidopsis thaliana*. CDC48 has close orthologues in animals, where it is also known as p97, valosin-containing protein (VCP), or transitional endoplasmic reticulum 94 (TER94) [4]. CDC48 orthologues have also been described in single-celled eukaryotes, such as amoebozoa and yeast (termed Cdc48) [6]. Herein we use CDC48/p97 to refer to features pertaining to all homologues.

**Fig. 1:**
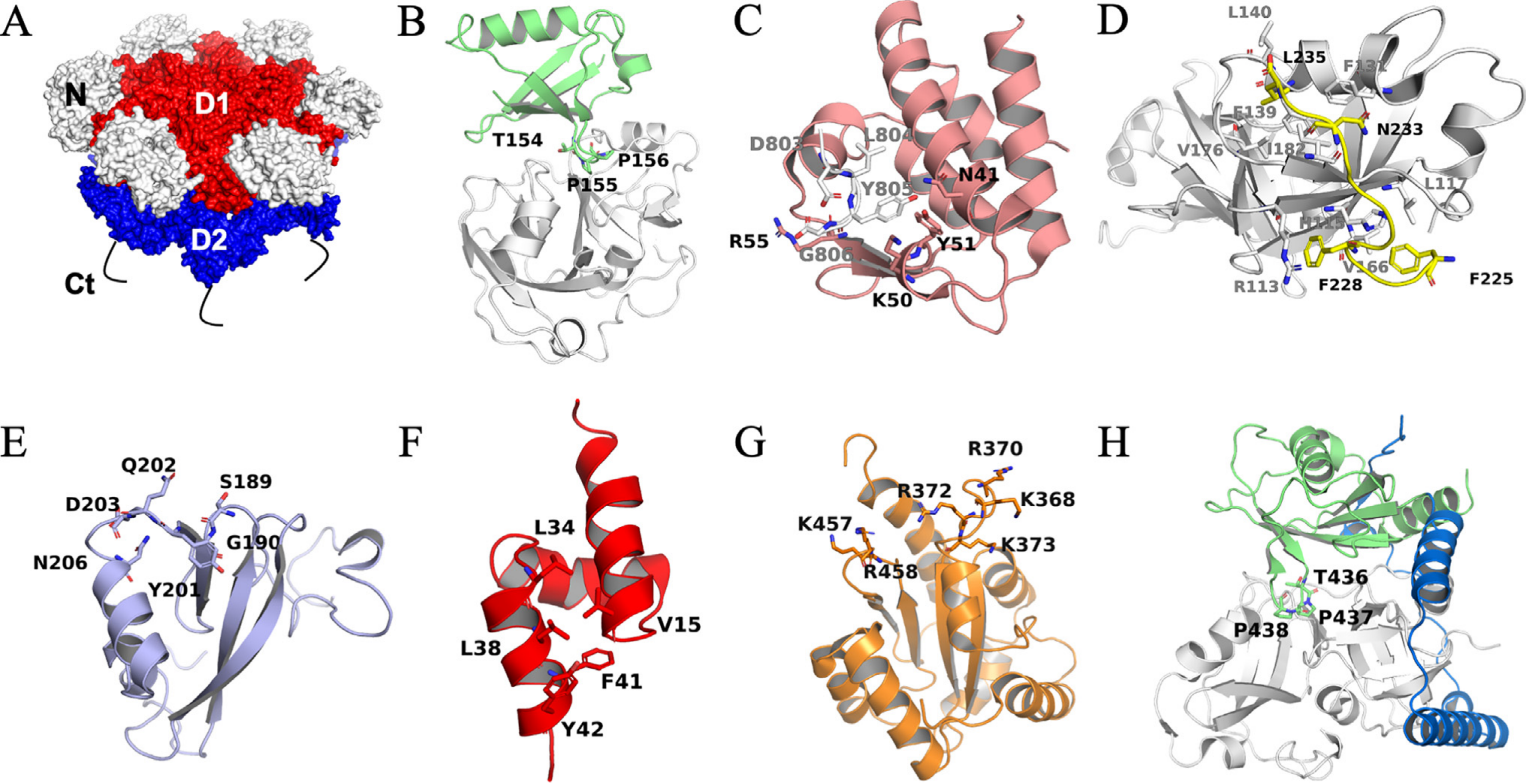
Structures of CDC48/p97 and PUX domains. A: Surface representation of *A. thaliana* CDC48A; homology model built with SWISS-MODEL [24], using the human p97 structure as a template (PDB 5FTN) [25]. The surfaces of the N, D1, and D2 domains are colour-coded, and the flexible C-terminal tail is indicated by a black line. Ct: C-terminal tail. B: The PUX1 UBX domain (green) in a complex with the N-terminal domain of p97 (p97-N) (white) (PDB 6HD0) [26]. C: Mouse PNGase PUB domain (pink) in complex with the p97 PIM motif (white) (PDB 2HPL) [27]. D: Human UFD1 SHP box (yellow) in complex with p97-N (white) (PDB 5B6C) [28]. E: Structural representation of conserved residues in the SEP domain of human p47 (PDB 1SS6) [29]. F: Rat p47 UBA domain (red) (PDB 1V92) [30]. Residues that may interact with ubiquitin are given. G: Human FAF1 UAS domain (orange) (PDB 2EC4) [31]. Residues of the positively charged surface patch are given. H: Structural representation of the UBX domain (green) and helical lariat (blue) of the human ASPL-C in complex with p97-N (white) (PDB 5IFS) [23]. Key residues for protein interactions are shown. (For interpretation of the references to colour in this figure legend, the reader is referred to the web version of this article.)

CDC48/p97 is composed of two stacked hexameric ATPase rings (formed by their N-terminal D1 and C-terminal D2 ATPase domains) (Fig. 1A) [7]. An additional ~190 residues N-terminal domain and a C-terminal stretch of ~75 flexible residues are used to bind cofactors and substrates [8], [9]. The flexible C-terminal tail also contributes to the regulation of the catalytic activity. The two ATPase rings form a central pore involved in substrate processing [4]. AAA ATPases use ATP hydrolysis for conformational changes, and CDC48/p97 uses the force generated through ATP hydrolysis to pull, extract, and unfold ubiquitylated or sumoylated proteins from the membrane, chromatin, or protein complexes [10]. This segregase activity is often, but not necessarily, linked with subsequent substrate degradation through the ubiquitin–proteasome system [11], [12]. Thus, CDC48/p97 contributes to protein homeostasis regulation, membrane fusion, vesicular trafficking, and chromatin-associated function [11].

CDC48 is an essential protein in plants and is in particular required when rapid cellular changes are needed, such as during reproduction, germination, growth, differentiation, and the immune response [13], [14]. In *A. thaliana*, CDC48A is involved in male gametophyte development [15], [16], centromere disassembly, the release of bulk ribosomal RNA genes in pollen for pollen-tube growth [17], and in the reshaping of lipid droplets to prime seeds for germination [18], [19]. CDC48A is also critical in shaping many aspects of sophisticated plant immune responses, where transcription and translation are enhanced, and in the degradation of immune receptors and of proteins from pathogens [14], [20], [21], [22].

CDC48/p97 orthologues in plants, yeast, and animals generally have more than 70% sequence identity, indicating that their function is essentially preserved. However, there are notable differences in the cofactors required for target selection and functional control of CDC48/p97 between plants and other species, reflecting their different physiologies.

## 2 Categories of CDC48/p97 cofactors

The multifunctionality of CDC48/p97 arises from their capacity to bind to different cofactors, of which more than 30 are currently known across plants, yeast, and animals [23]. These cofactors can be divided into the following three classes based on their functions and domains: first, noncatalytic substrate-recruiting cofactors that contain one domain for binding to CDC48/p97 and another domain for binding to ubiquitin or small ubiquitin-like modifier (SUMO)-modified substrates. Prominent examples of these cofactors include ubiquitin-associated and ubiquitin regulatory X (UBA-UBX) proteins, and the complex formed by the ubiquitin recognition factor in the endoplasmic reticulum (ER)-associated degradation protein 1 (Ufd1) with the nuclear protein localization protein 4 homologue (Npl4). The second class includes substrate-processing cofactors, such as ubiquitin E3 ligases and deubiquitinases. The third class consists of cofactors that regulate the function of CDC48/p97 through the disruption of hexamers, post-translational modifications (PTMs), or sequestering [4].

These diverse cofactors bind to only a small number of sites on CDC48/p97. The majority of the cofactors bind to the N-terminal domain, either through a UBX or UBX-like (UBXL) domain or through specific linear binding motifs, such as the SHP binding motif [4]. The resulting competition between cofactors may help commit CDC48/p97 to specific functions. Whereas the CDC48/p97 cofactors are relatively well understood in mammals and yeast, their identification and characterization in plants has been very sporadic.

### 2.1 The PUX protein domains

The *Arabidopsis* genome contains at least 16 UBX-containing proteins that are collectively called the plant ubiquitin regulatory X domain-containing proteins (PUX) [32]; approximately half of these have been investigated to date [16], [18], [19], [32], [33], [34]. The identified functions already provide an indication for the many ways through which PUX proteins can control CDC48A. Several members function as canonical adaptors to recruit the proteolytic activity of CDC48 to diverse cellular loci and organelles, whereas others have evolved different approaches to inhibit CDC48 function. Additionally, although some PUX proteins recruit ubiquitinated proteins to CDC48, others mediate the degradation of nonfunctional CDC48 [34]. Herein, we first provide an overview of the individual domains and motifs of PUX proteins and subsequently summarise the emerging knowledge of how the combination of these domains produces specific biological functions in plants.

**Fig. 2:**
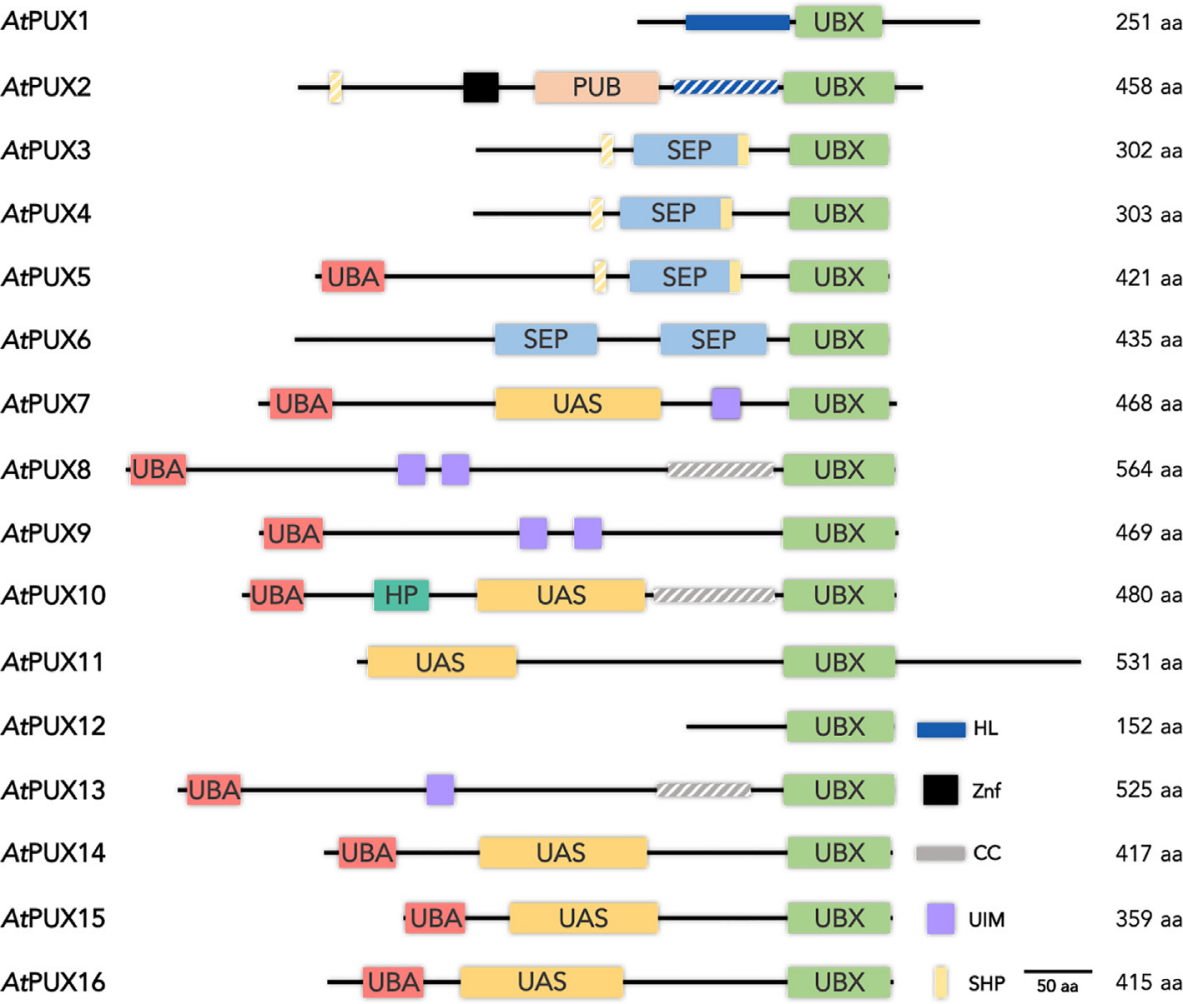
Domain architecture and classification of UBX domain–containing proteins. Folded protein domains are shown boxed with domain names. UBA: Ubiquitin-associated; PUB: Peptide: N-glycanase/UBA or UBX-containing proteins; SEP: *Saccharomyces cerevisiae*, *Drosophila melanogaster*, eyes closed gene and vertebrate p47; UAS: Ubiquitin-associating; and UBX: Ubiquitin regulatory; HP: Hydrophobic patch. Linear functional motifs are colour coded: HL: Helical lariat; Znf: Zinc finger; CC: Coiled-coil region; UIM: Ubiquitin-interacting motif; SHP box: linear motif derived from Shp1p [43]; aa: amino acids. The scale bar shows the approximate length of 50 aa. Putative motifs or structural elements identified in this study are shown in stripes.

PUX proteins are a very heterogeneous family. Their sequences range from 150 to more than 500 amino acids in length and contain various combinations of a dozen interaction domains and sequence motifs linked by unstructured regions (Fig. 2). The only common feature is the C-terminal UBX domain. Three-dimensional structures have not yet been determined for other domains, and their cellular ligands and functions are often incompletely understood.

### 2.2 The UBX domain

The UBX domain is the general binding domain that enables PUX proteins to associate with CDC48 [35]. UBX is the only PUX domain for which a three-dimensional structure has been determined, namely for *A. thaliana* PUX1 bound to the human p97 [26]. The PUX1 UBX domain adopts a ubiquitin-like fold. Akin to the UBX domains of rat p47 and human FAF1, PUX1 UBX binds to the N-terminal domain of p97 (p97-N) [36], [37], [38]. The interaction is mediated by a loop between the UBX ß-strands 3 and 4 (termed the S3-S4 loop) that contains a crucial proline residue. This motif inserts into a hydrophobic pocket of p97-N (Fig. 1B) [36], [38]. The S3-S4 loop motif is conserved in PUX proteins suggesting that they bind to CDC48-N, with the notable exception of PUX2 (Supplementary Figure 1A). However, to date the association with CDC48 has only been confirmed experimentally for the UBX domains of PUX1, 7, and 10 [16], [18], [19], [32].

### 2.3 The PUB domain

The ~ 100-residue PUB domain is the only domain from UBX-containing proteins known to interact with the p97 C-terminal tail (p97-C) [39]. In *Arabidopsis*, the PUB domain is only present in PUX2, which has a poorly understood molecular function. However, PUB domains found in human proteins (PNGase, UBXD1, and HOIP) are reportedly bound to p97 [40], [41], [42]. Analysis of the interactions of PNGase, UBXD1, and HOIP with their respective ligands revealed a common PUB interaction motif (PIM). In p97, this PIM is formed by the four residues D^803^LYG, located at the end of its C-terminal tail, and it associates with residues N^41^, K^50^, Y^51^, and R^55^ in the human PNGase PUB domain (Fig. 1C) [27], [40], [42]. The PIM-binding surface patch is conserved in the PUX2 PUB domain; furthermore, a putative PIM, based on our sequence comparison, is present in CDC48A-C (residues D^806^LYN, Supplementary Figure 1B), suggesting that the PUX2 PUB domain also binds to CDC48A-C.

### 2.4 The SHP box

The SHP box is a 10 amino acid motif with the consensus sequence F-x-G-x-G-x-x-*h*, where x is any residue and *h* is a hydrophobic residue, usually leucine. The SHP box, previously also called binding site 1 [44], is the only other element in UBX-containing proteins that interacts with the CDC48/p97-N domain [44], [45], [46]. This motif was first identified in several yeast proteins, including Dfm1, Shp1, and Ufd1 [43]. Nuclear magnetic resonance (NMR) and X-ray crystallographic analyses showed that the SHP box binds to human p97-N. However, these studies disagreed on whether the SHP box and the UBX domain could bind simultaneously to the same CDC48/p97-N domain [28], [47]. Upon binding to p97, the human UFD1 SHP box adopts an elongated structure [28]. When binding with p97, UFD1 uses F^225^ aside from the F^228^, N^233^, and L^235^ residues of the SHP sequence (Fig. 1D) [28]. These residues are conserved in PUX proteins, except for F^225^, which is replaced by a positively charged residue (Supplementary Figure 1C). Whether this replacement will abolish the interaction of the SHP box with CDC48A remains to be established. PUX2, 3, 4, and 5 contain additional putative SHP box motifs in their sequences with unknown significance (Fig. 2, Supplementary Figure 1C).

### 2.5 The SEP domain

The SEP domain was named according to the following three protein orthologues in which it was identified: Shp1 from *S. cerevisiae*, eyes closed from *D. melanogaster,* and human p47 [29]. Four PUX proteins contain SEP domains. Three of them (PUX3, 4, and 5) also harbour one or possibly two SHP box(es) (Fig. 2), suggesting that the SEP domain and SHP box act as a functional entity in these members. The human p47 SEP domain was suggested to function as a cysteine protease inhibitor based on the structural similarity of its S1-S2 and S3-H1 loops with the corresponding loops in cystatins and stefins (Fig. 1E) [29]. These loop residues are also conserved in the SEP domains of PUX3, 4, 5, and 6 (Supplementary Figure 1D). Therefore, PUX SEP domains may also function as protease inhibitors, but possibly with altered specificity and/or affinity. However, this functional annotation is not the only one that has been proposed; the SEP domain of human p37, in combination with a helix located upstream of SEP, can reportedly mediate a ubiquitin-independent degradation of substrates by p97 [48], [49]. A helical region of similar length is also predicted for PUX3, 4, and 5 (Supplementary Figure 1C). Finally, the SEP–SHP fragment of human p47 was shown to self-associate [30]. This self-association was disrupted when the p47 SEP-UBX fragment bound to p97-N [30], suggesting an autoregulatory mechanism resulting from the competition between intra- and intermolecular interactions. PUX3, 4, and 5 were shown to target CDC48 to the nucleoskeleton [33]. However, the mechanism through which the SEP–SHP module contributes to this function has not yet been established. PUX6, which contains a tandem SEP module without SHP motifs, was not found to localise to the nucleoskeleton [33]. Hence, the PUX6 SEP domain might perform a different function.

### 2.6 The UBA domain

The UBA domain was identified through sequence analysis of proteins that may be involved in the ubiquitin–proteasome pathway [50]. The UBA domain consists of approximately 40 amino acids and forms a three-helix bundle, although only a few residues are highly conserved (Fig. 1F) [51], [52]. Even though ubiquitin-binding is the general feature proposed for UBA domains [50], two different cellular functions have been ascribed to them: degradation and stabilisation. Degradation-class UBA domains interact with multi-ubiquitinated substrates and deliver these to the proteasome with the help of the UBX domain. Stabilising UBA domains block the elongation of the ubiquitin chain by capping the ubiquitin anchored on the substrate [53], [54]. A comparison between the UBA domains of the Ucp1 and Ddi1 orthologs from fission and brewer’s yeasts, respectively, showed a different preference for mono- vs. tetraubiquitin, indicating a specialisation of the UBA domains towards degradation or stabilisation [55], [56].

Nine of the 16 PUX proteins contain a UBA domain at their N-terminus (Fig. 2), suggesting that also PUX proteins may have UBA domains specialised for degradation or stabilisation. An interaction with ubiquitin was confirmed experimentally for the UBA domains of PUX7 and PUX10 [16], [18]. Although the cellular role of PUX10 in guiding active CDC48A to extract proteins from vesicles supports a ‘degradation mode’ of the PUX10 UBA domain [18], [19], the exact role of this and other PUX family UBA domains remains to be clarified.

### 2.7 The UAS domain

The ~ 110 residue UAS domain belongs to the thioredoxin-like superfamily [57], but lacks the C-x-x-C motif that conveys the reductional capability to thioredoxin. Rather, the analysis of human UAS domains from FAF1 and FAF2 (also called UBXD8) identified a fatty acid (FA) sensor function [31]. The UAS domain can mediate the polymerisation of FAF2, which is a sensor for long-chain unsaturated FAs. Two positively charged patches on the UAS domain of both FAF1 and FAF2 are essential for promoting FA-induced polymerisation (Fig. 1G) [31].

Six *Arabidopsis* PUX proteins contain a UAS domain (PUX7, 10, 11, 14, 15, and 16) (Fig. 2). Our structural modelling suggests that they display positively charged surface patches that resemble that of FAF2 UAS, except for the missing positive residues in the S3-S4 loop of PUX11 and PUX15 (Supplementary Figure 2A). The possibility that PUX UAS domains are sensors of different types of FA will need to be experimentally assessed.

### 2.8 The UIM motif

The ubiquitin-interacting motif (UIM) is a short motif of approximately 20 amino acids with the consensus *h*-x-x-A-x-x-x-S-x-x-*A^c^*, where *A^c^* denotes an acidic residue [58]. The UIM motif was first identified as a ubiquitin-interacting region in the human S5a subunit of the 19S regulator complex [59]. Subsequent mutational analyses confirmed the importance of conserved UIM residues for ubiquitin binding [60], [61]. A subset of UIM motifs can bind to ATG8, a ubiquitin-fold protein implicated in autophagy [34]. PUX7, 8, 9 and 13 all bind to ATG8 with their UIM motifs (containing the *A^c^*-*A^c^*-x-x-*h*-x-x-A-*h*-x-x-S-x-x-*A^c^* consensus) to mediate the autophagy and clearance of inactive CDC48 [34].

### 2.9 Other domains and motifs

Several PUX members harbour additional functional regions. The N-terminal region of the PUX1 UBX domain displays secondary structure and physicochemical features similar to the helical latch, or lariat, of the human UBX domain-containing protein alveolar soft part sarcoma locus (ASPL) (Fig. 1H). Structural studies of ASPL show that the 60 residues N-terminal of its UBX domain latch around p97 (Fig. 1H), thereby competing with p97′s self-association and disrupting the p97 hexamer [23]. The hexamer-disrupting function of this lariat appears preserved in PUX1 [26], [32].

A zinc finger domain of unknown function has been identified in PUX2 by sequence analysis [62]. Our homology modelling suggested that this domain displays sequence and structural features compatible with the type 4 ubiquitin-binding zinc finger of the human Rad18 protein (Supplementary Figure 2B) [63], [64]. Hence we speculate that PUX2 might use this function to recognise ubiquitinated ligands.

Sequence analysis identified elongated helical regions just upstream of the UBX domains of PUX8, 10, and 13 (Fig. 2). Unlike the helical lariat of PUX1, these regions have sequences compatible with dimeric coiled-coil structures [65], suggesting that these regions mediate (homo) oligomerisation.

PUX10 uses a ~ 20-residue hydrophobic hairpin sequence to localise to the phospholipid monolayer that envelopes lipid droplets (LDs) [18], [19]. This sequence is only present in PUX10 within the PUX family, but similar hairpins are used by several LD-localised proteins, such as oleosins which are the major LD-associated proteins in plant seeds.

Additionally, the long unstructured PUX family protein regions are likely to contain additional linear interaction motifs and sites for PTMs, as suggested, for example, by large-scale phosphoproteome profiling [66], [67]. These sites require further experimental investigations.

## 3 Controlling CDC48 activity

CDC48 is a ubiquitous segregase present throughout the cell. In plants, the activity of CDC48 is largely controlled and directed by PUX proteins. The emerging structural, functional, and biological knowledge starts to reveal the many ways by which individual PUX family members control CDC48.

PUX1 has been shown to inactivate CDC48A by dissociating the CDC48 hexamer [26], [32], [68]. For this, the PUX1 UBX domain binds to the N-terminal of CDC48A, as shown by the hybrid structure of the PUX1 UBX domain in a complex with human p97. The capability of PUX1 to dissociate the CDC48 hexamer originates from the N-terminal helical lariat of the PUX1 UBX domain that wraps around the CDC48 monomer (Fig. 1H) [26], [32], [68]. Lacking additional domains and known functional motifs, the major role of PUX1 appears to be to prevent CDC48A from assembling.

PUX2 is a compatibility factor for powdery mildew (PM) as mutations in this protein inhibit the growth of PM in *A. thaliana* [62]. This effect was linked to PUX2′s capability to decrease basal mesophyll cell ploidy, thus increasing endoreduplication, and hence metabolic capacity, at the fungal feeding site [69]. PUX2 may influence cell ploidy by affecting cell expansion and differentiation via its interaction with CDC48 at the cell division plane [32], [69]. PUX2 is the only PUX protein that has a PUB domain, which binds to the C-terminal of CDC48A. PUX2 also has an atypical UBX domain, where the S3-S4 loop motif is unusually long (Supplementary Figure 1A), raising the possibility that it does not bind to CDC48. These features suggest that PUX2 is an atypical member that interacts with CDC48 via the PUB domain instead of the UBX domain, which is freed and modified for different associations. Its zinc finger domain might functionally replace the UBA domain for ubiquitin binding. Intriguingly, the region preceding the PUX2 UBX domain displays sequence and secondary structure features reminiscent of the N-terminal lariat of PUX1 and human ASPL. It remains to be determined how these unusual features combine to produce the biological function of PUX2.

PUX3, 4, and 5 were shown to recruit CDC48 to the nucleoskeleton underneath the inner nuclear membrane (INM) [33]. Similar to PUX1, these three PUX proteins negatively regulate protein degradation by CDC48. However, the exact mechanism remains unclear. Their UBX domains appear to be canonical CDC48-interacting domains without indications of an N-terminal latch found in PUX1, inferring that the UBX domains of these three PUX proteins recruit CDC48 without disrupting the hexamer. PUX3, 4, and 5 have a SEP domain and SHP motifs, suggesting that these regions mediate the interactions with the nucleoskeleton, possibly in a ubiquitin-independent way, thereby targeting CDC48 to these locations. Whether or how the SEP domain and SHP motifs influence CDC48 activity is currently unknown. It has been suggested that PUX3, 4, and 5 inhibit the function of CDC48 directly through an unknown mechanism or indirectly by degrading a ubiquitin ligase that targets the INM proteins [33]. The presence of a UBA domain in PUX5, but not in PUX3 and 4, might indicate a slightly different function of PUX5 by allowing it to recruit ubiquitinated substrates.

PUX7, 8, 9, and 13 mediate the autophagic clearance of inactive CDC48. Autophagosomes emerge from the ER as cup-shaped phagophores decorated by the ubiquitin-fold protein ATG8 [34]. PUX7, 8, 9, and 13 bind to ATG8 and CDC48 via their UIM motif and UBX domain, respectively [34]. Although PUX7/8/9/13 are continuously turned over by autophagy, and PUX7/8/9/13 and CDC48 are turned over during nitrogen starvation in *Arabidopsis*, these PUX proteins only mediate autophagic clearance of CDC48 that has been first rendered nonfunctional through small-molecule inhibitors or through deleterious mutations [34]. How the PUX proteins specifically identify inactive CDC48 remains to be determined. However, given that all four PUX members have an N-terminal UBA domain, the involvement of ubiquitination is a possibility. PUX7, 8, 9, and 13 jointly contribute to full clearance of inactive CDC48, inferring functional redundancy. Yet, PUX7 also has a central UAS domain and hence might have specific additional functions.

PUX10 recruits CDC48 to LDs to regulate the protein composition of these ER-derived organelles. However, conversely to PUX3/4/5 in INM, CDC48 recruited by PUX10 is catalytically active and extracts ubiquitinated proteins from the LDs for LD-associated degradation (LDAD) [18], [19]. PUX10 uses its hydrophobic hairpin to localise to LDs. There, it functions as an adaptor protein that uses the UBX domain to bind to CDC48-N and the UBA domain to recruit active CDC48 to ubiquitinated LD proteins to catalyse their extraction from LDs.

The function of the PUX10 UAS domain is currently unknown, but the FA-binding activity observed in human UAS domains would provide a function matching the LD environment. By controlling the LD-associated degradation (LDAD) of ubiquitinated oleosins and possibly other LD surface proteins, PUX10 regulates the LD size and therefore availability of the lipids stored within it. Thus, PUX10 was shown to regulate the turnover of LDs during seed germination and embryogenesis in *A. thaliana* and may also regulate LDs in tobacco pollen tubes [18], [19]. Additional localisations of PUX10 to the membranes of chloroplasts and the ER suggest PUX10 might also have roles in chloroplast-associated degradation (CHLORAD) and ER-associated degradation (ERAD) [18].

## 4 Conclusion

By translocating and/or eliminating proteins from various locations in the cell, CDC48/p97 serves as a fast-response mechanism to changing conditions and assures a healthy proteome in plants, animals, and single-celled eukaryotes. CDC48/p97 proteins are highly conserved in their sequence and segregase activity. The functional versatility of CDC48/p97 arises from its combination with an arsenal of adaptor proteins that control both the activity and targets of CDC48/p97.

PUX proteins emerge as adaptor proteins that target CDC48 to several organelles, thereby controlling membrane-associated protein translocation and degradation. This assures normal organellar homeostasis and allows adaptation to developmental and environmental cues. CDC48-mediated protein degradation has been associated with CHLORAD, LDAD, ERAD, INM, and autophagy in plants [18], [19], [33], [34], [70], [71], [72]. All these protein degradation mechanisms were shown to involve PUX proteins, although the evidence linking PUX proteins to CHLORAD and ERAD remains weak [18], [19].

Further resolving the pieces of the PUX puzzle requires a combination of structural and functional approaches. At the domain and motif levels, we require a better understanding of the cellular targets of the individual PUX domains and the experimental determination of structures of PUX family domains, both when alone and bound to their targets. Additional efforts need to be directed towards identifying higher-level regulatory mechanisms. In plants, CDC48A is controlled through PTMs such as S-nitrosation in tobacco [73] and phosphorylation in *Arabidopsis* [15]. However, the effects of PTMs on PUX proteins and their association with CDC48 remain unexplored. Additional understudied mechanisms include conformational dynamics and self-associations that may collectively regulate PUX proteins and their ligands. Furthermore, it is important to elucidate whether and how specific combinatorial effects are produced by the simultaneous interaction of one CDC48 hexamer with more than one type of adaptor protein. For example, the combination of different PUX proteins or the combination of PUX proteins with other factors, such as adaptors of the Npl4/Ufd1 families, may each give rise to specifically tuned responses [74]. At the organismal level, it is noteworthy that the biological function of PUX11, 12, 14, 15, and 16 still has not been investigated.

Owing to their relatively small size and high flexibility, establishing the structure–function relationship of PUX proteins will require integrative and innovative approaches. We anticipate that the combination of computational approaches, high- and low-resolution structural methods, and functional *in vitro* and *in planta* assays will be necessary to appreciate the functional palette of PUX proteins. An enhanced understanding of how the PUX proteins allow plants to maintain proteostasis and to adapt to changing conditions may inspire novel genetic approaches to enhance desired features in agriculturally important species.

## CRediT authorship contribution statement

**Junrui Zhang:** Conceptualization, Visualization, Investigation, Writing - original draft, Writing - review & editing. **Alexandra I. Vancea:** Conceptualization, Visualization, Writing - original draft, Writing - review & editing. **Umar F. Shahul Hameed:** Conceptualization, Writing - original draft, Writing - review & editing. **Stefan T. Arold:** Supervision, Funding acquisition, Conceptualization, Investigation, Visualization, Writing - original draft, Writing - review & editing.

## Declaration of Competing Interest

The authors declare that they have no known competing financial interests or personal relationships that could have appeared to influence the work reported in this paper.
