# Supplementary material for: Versatile control of the CDC48 segregase by the plant UBX-containing (PUX) proteins
Source: Comput Struct Biotechnol J. 2021 May 14;19:3125–32. doi: 10.1016/j.csbj.2021.05.025 (PMC8181520; doi:10.1016/j.csbj.2021.05.025)
Supplement: Supplementary data 1 [file mmc1.docx]

**Supplementary Figures**


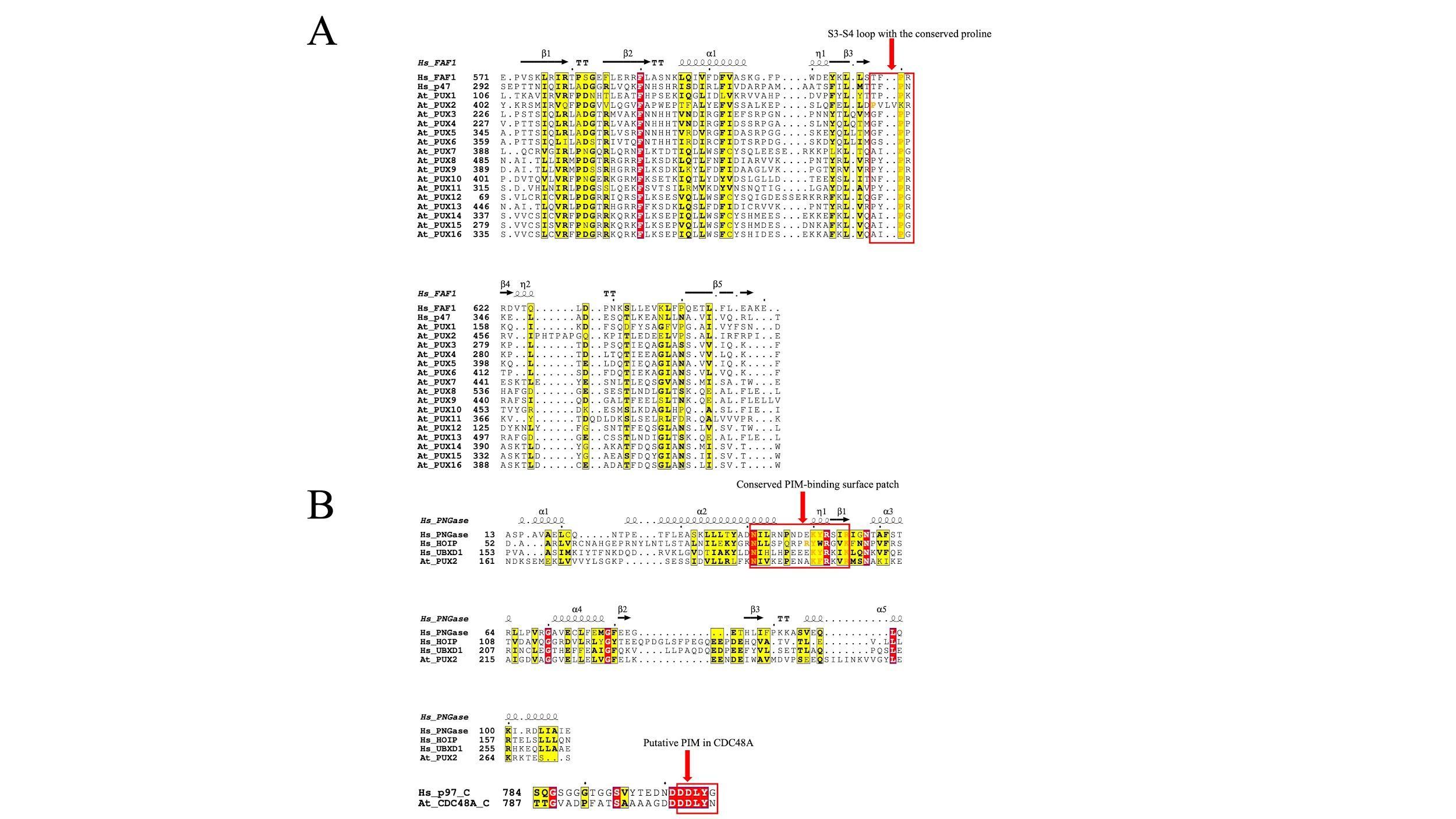


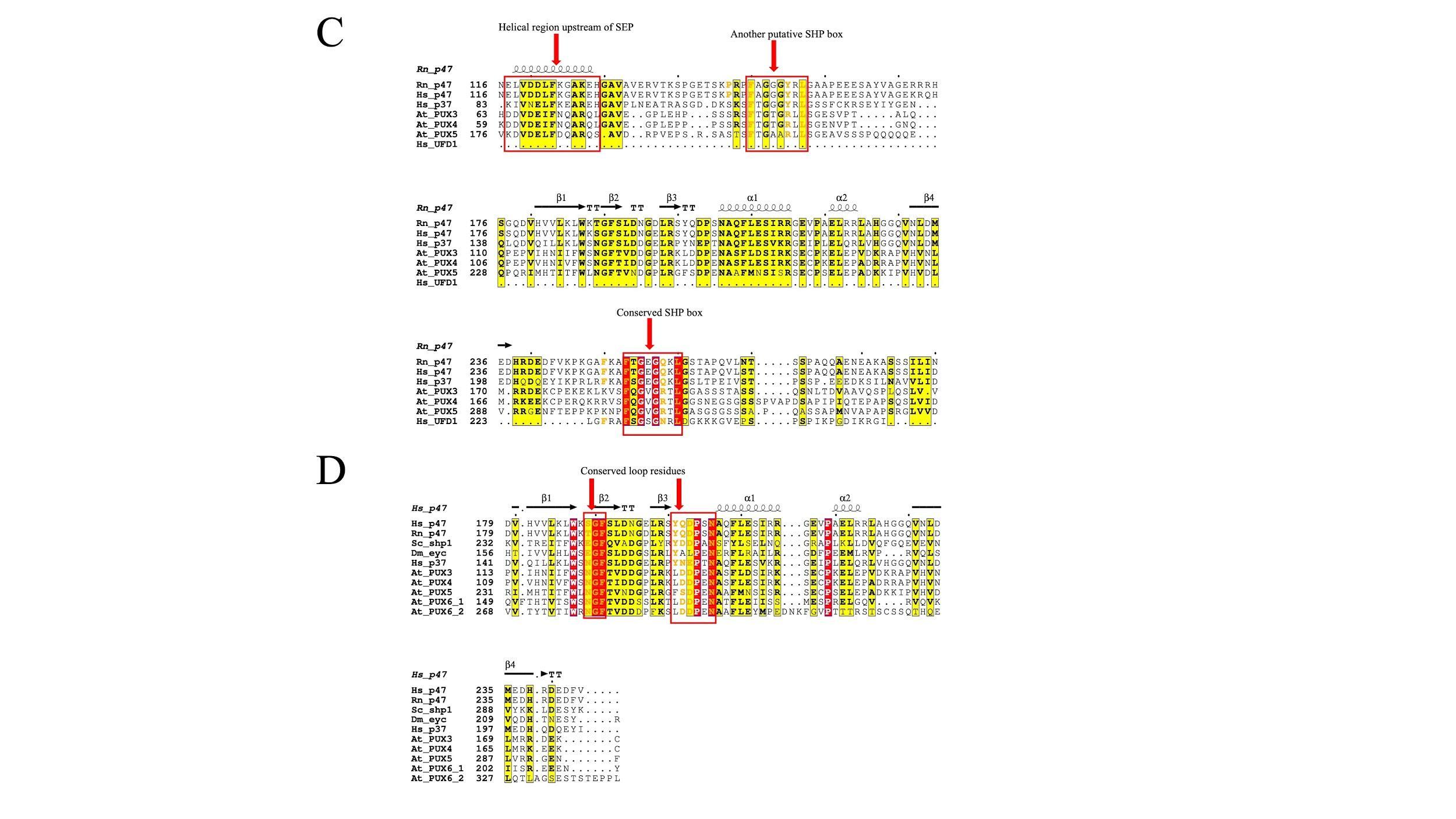


**Supplementary Figure 1: Sequence alignment of PUX domains.** Sequence alignment is carried out using Tcoffee [1], and the figure is made using ESPript 3.0 [2]. Key residues are additionally coloured in orange. (*Arabidopsis thaliana*: At; *Homo sapiens*: Hs; *Rattus norvegicus*: Rn; *Saccharomyces cerevisiae*: Sc; *Drosophila melanogaster*: Dm) **A**: Sequence alignment of the UBX domain in all PUX proteins. **B**: Sequence alignment of the PUB domain and the PIM motif. **C**: Sequence alignment of the SHP box together with the SEP domain. **D**: Sequence alignment of the SEP domain


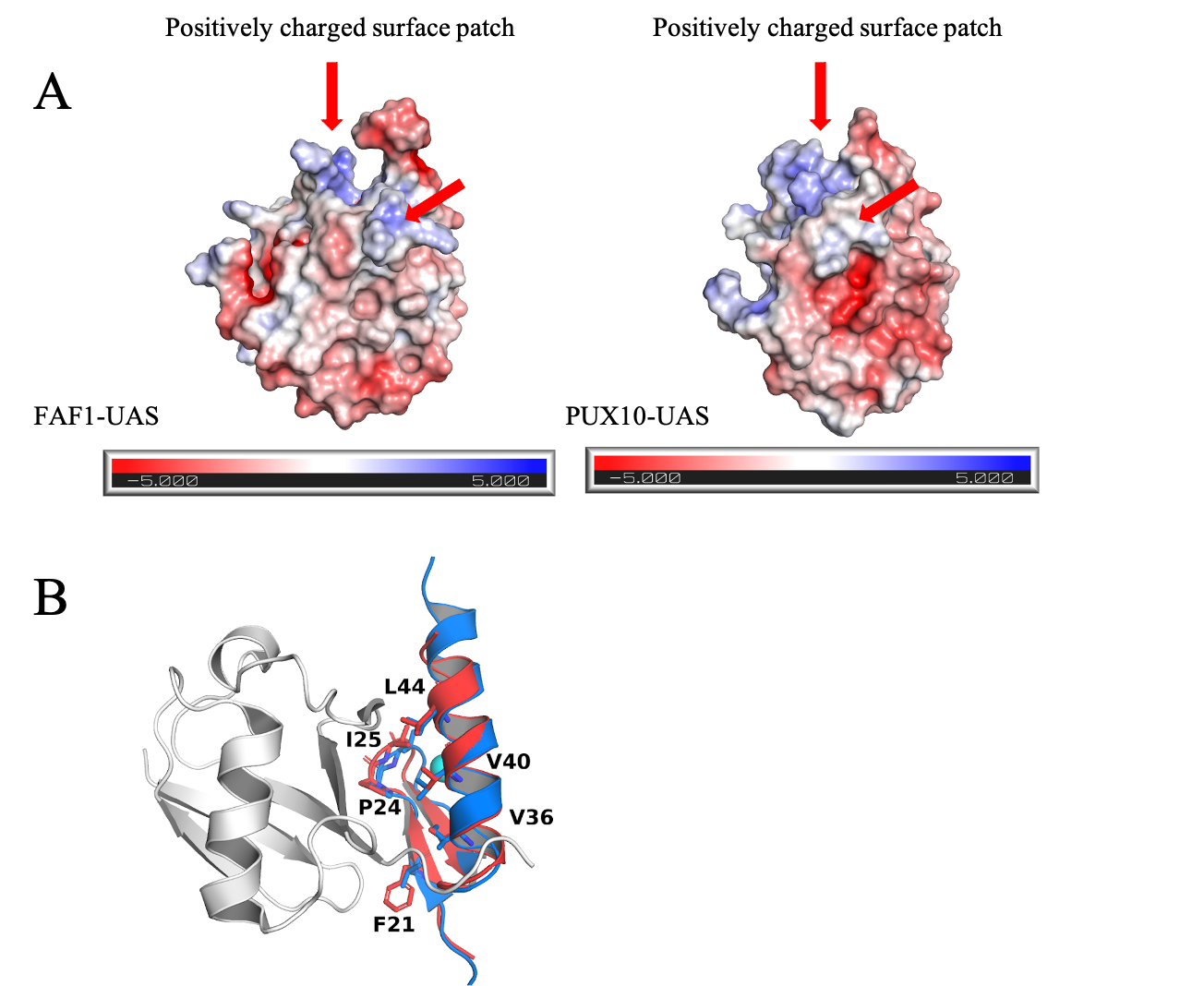


**Supplementary Figure 2: Supplementary structural information. A**: The electrostatic surface of the UAS domain, coloured from red (negative) to blue (positive). *Left:* Positively charged surface patches on the FAF1 UAS domain are indicated by arrows (PDB 2EC4) [31]; *Right:* Corresponding positively charged surface patches of the PUX10 UAS domain are indicated. The structural model was built with SWISS-MODEL [24]. **B**: Structural modelling of the putative interaction between the PUX2 zinc finger and ubiquitin. The structure of the PUX2 zinc finger (red) was built with SWISS-MODEL, using the type 4 ubiquitin-binding zinc finger (UBZ) of the human Rad18 protein (blue) in a complex with ubiquitin (white) as a template (PDB 5VF0) [24, 64]. Key residues for the ubiquitin association are shown and the zinc ion is coloured in cyan.

**References:**

[1]: Notredame C., Higgins D.G., Heringa J. T-Coffee: A novel method for fast and accurate multiple sequence alignment. J Mol Biol 2000;302:205–217. doi:10.1006/jmbi.2000.4042.

[2]: Robert X., Gouet P. Deciphering key features in protein structures with the new ENDscript server. Nucleic Acids Res 2014;42:W320–W324. doi:10.1093/nar/gku316.
